# Use of posturography in patients with ankylosing spondylitis: A systematic review

**DOI:** 10.4102/sajp.v80i1.1953

**Published:** 2024-05-14

**Authors:** Caroline F.O. Silva, Karen Obara, Luana Paixão, Eduarda H. Santos, Amanda I.Z. Santos, Jefferson R. Cardoso

**Affiliations:** 1 Department of Physical Therapy, Universidade Estadual de Londrina, Londrina, Brazil; 2 Laboratory of Biomechanics and Clinical Epidemiology, PAIFIT Research Group, Universidade Estadual de Londrina, Londrina, Brazil

**Keywords:** ankylosing spondylitis, postural control, force platform, biomechanics, spondyloarthropathy

## Abstract

**Background:**

Ankylosing spondylitis (AS) is characterised as a chronic inflammatory disease of the axial skeleton. The force platform is an option for performing the postural assessment of these individuals.

**Objectives:**

To review and evaluate the behaviour of the centre of pressure (CoP) variables during the postural control examination in patients with AS compared to a control group.

**Method:**

A systematic review, registered in PROSPERO, that followed the PRISMA Statement. A search was carried out in the following databases: Medline, Web of Science, Embase, Scopus, and Scielo, from 1945 to 2023. Studies were selected that aimed to understand the use of the force platform for the assessment of postural control. The risk of bias assessment was performed using the AXIS tool.

**Results:**

Five studies were included, with a total of 247 participants. The assessment of risk of bias presented high scores in the AXIS tool. Patients with a diagnosis of AS presented increased thoracic kyphosis in most of the studies, as well as large displacements in the anteroposterior (AP) and mediolateral (ML) directions, and altered total mean velocity (TMV) and frequency, indicating worse postural stability. Regarding the functional status, the most used questionnaires were the Bath Ankylosing Spondylitis Functional Index (BASFI), Bath Ankylosing Spondylitis Metrology Index (BASMI) and Bath Ankylosing Disease Activity Index (BASDAI).

**Conclusion:**

Patients with ankylosing spondylitis present postural instability, verified by means of higher values of centre of posture variables.

**Clinical implications:**

Individuals with ankylosing spondylitis presented postural instability and balance deficit. Therefore, exercises for balance training and postural control are essential in the clinical management of these patients.

## Introduction

Ankylosing spondylitis (AS) is an inflammatory and chronic disease of the axial skeleton that occurs frequently between 20 and 40 years of age. Intragroup differences are observed in individuals with positive HLA-B27 during the clinical course, and an insidious onset with slow progression and increased remissions is also noted (Atar & Askin 2020; Nam et al. 2022; Yildirim & Yildirim 2015). It may be diagnosed using the following radiographic findings: the presence of asymptomatic sacroiliitis, low back pain for > 3 months and positive HLA-B27 (Ritchlin & Adamopoulos 2021).

The main characteristics of AS are sacroiliitis, enthesitis and vertebral fusion propensity, which lead to the common symptoms of chronic low back pain and progressive spinal stiffness (Nam et al. 2022; Ward et al. 2016). This last clinical aspect, together with the adoption of an antalgic position by the individual, leads to decreased mobility and flexibility, resulting in an anteriorised posture accompanied by a change at the centre of mass (CM) (Çinar et al. 2016). This can lead to an increase in the prevalence of falls, comprising 34.7% for patients with AS (El Miedany et al. 2010). The literature review by Pompeu et al. (2012) corroborated these statements and added that inadequate functioning of mechanoreceptors, due to enthesitis and muscle weakness, suppressed the rapid muscle response and, therefore, can impair the postural balance.

Postural control or balance is an adaptation of posture with changes at the CM during static and dynamic postures, keeping it within the base of support with minimal oscillations. This requires precise coordination of the visual, auditory, proprioceptive, neuromuscular and central nervous system (De Nunzio et al. 2015; Uckun & Sezer 2017). However, the increasing inflammatory process of AS results in neuromuscular and proprioceptive system deficits, thus causing some anatomical changes such as lumbar lordosis rectification, cervical lordosis inversion and increased thoracic kyphosis. This last condition is one of the factors responsible for destabilising the CM. Owing to these factors, patients perform some compensation to avoid CM changes, such as hip extension, posteriorisation of the pelvis, knee flexion and ankle plantar flexion (Sawacha et al. 2012).

For postural control assessment, the force platform is the most used equipment, which consists of plates equipped with force sensors (a load cell or a piezoelectric system) that measure the force and torque components (Duarte & Freitas 2010). The technique used to verify body sway in the force platform is known as posturography. Static posturography, specifically, might be useful in monitoring the disease severity, because the standing postural control is significantly altered in patients with AS (De Nunzio et al. 2015; Vergara et al. 2012). The most used measure to assess posture is the centre of pressure (CoP), which represents the ground reaction force vector that can be measured by a force platform (Chen et al. 2021). In other words, the CoP is the result of muscle activation and body weight, which exert force through the feet on the platform and provide CoP signals, encompassing the oscillations in the anteroposterior (AP) and mediolateral (ML) directions (Chen et al. 2021; De Nunzio et al. 2015). Our study attempted to answer this question using the PECO (Population, Exposure, Comparison and Outcome) mnemonic: Does AS (P) negatively affect the CoP values (O) during posturography analysis (E) when compared to healthy individuals (C)? Our systematic review of observational studies aimed to assess and evaluate the characteristics of CoP variables, for example, total oscillation displacement (TOD), area, total mean velocity (TMV) and its amplitude and variability (AP and ML), during the postural control examination using the force platform.

## Method

Our systematic review without a meta-analysis was registered in PROSPERO (International Register of Systematic Reviews – #CRD 42022363337) and followed the recommendations of the Cochrane Collaboration (Higgins et al. 2022) and the PRISMA Statement (Page et al. 2021).

### Eligibility criteria

The included studies encompassed posturography in patients with AS using the force platform and compared it with healthy participants. Those who used other types of equipment/method to assess postural control, such as the Berg Scale and pedobarography, were excluded. No age limits were set for the participants.

### Search strategy

Databases of Medline (Medical Literature Analysis and Retrieval System Online, 1950–2023), Web of Science (1945–2023), Embase (Excerpta Medica Database, 1947–2023), Scopus (1996–2023) and SciELO (Scientific Electronic Library Online, 1998–2023) were searched up to February 2023. Two experienced reviewers formulated the search strategy, and a third member of the team resolved possible disagreements. No language restriction was set during the search strategy.

The following keywords were combined with Boolean operators (AND/OR): ankylosing spondylitis, spondyloarthritis, posture, posture control, postural sway, postural analysis, posturography and balance.

### Centre of pressure variables

The CoP variables considered were divided into global and structural parameters. The global class measures the extent of CoP oscillation relative to time and frequency. The structural parameters identify patterns of oscillation and relate them to muscle control.

Among the elements included in the global analysis are length of the CoP trajectory in the AP and ML, designated full displacement; area, which brings the area of oscillation of the CoP in the AP and ML directions in relation to a point in the centre of the force platform; and the TMV. In the structural analysis, it encompasses the amplitude of oscillation in both AP and ML directions. In addition, within the amplitude analysis, the distance between two consecutive points in the CoP trajectory was evaluated, where the greater distance represented a slow and low efficiency of motor control (De Nunzio et al. 2015; Duarte & Freitas 2010).

### Selection and data collection

Two independent reviewers performed the selection, inclusion of studies and data extraction and followed the eligibility criteria of this review and recommendations of the PRISMA Statement (Page et al. 2021).

### Risk of bias assessment

The studies were assessed for risk of bias by two independent reviewers, and any disagreements between them were discussed with a third reviewer. The modified cross-sectional assessment tool (AXIS) (Downes et al. 2016) was used, and the items were clear objectives, appropriate design, adequate and clearly defined sample size, measured result, adequate instruments, clear statistics, determination of significance and sufficiently reproducible method. A modification was made to suit the type of study included; thus, the questions 5, 7, 13, 14 and 15 of the AXIS tool were withdrawn (mostly related to non-respondents). Items were rated as high risk, uncertain risk and low risk of bias (Downes et al. 2016).

### Data analysis

The characteristics of the included and excluded studies are presented descriptively.

### Ethical considerations

Our study consists of secondary research; thus, ethical approval was not required for our systematic review.

## Results

### Study selection

**FIGURE 1:**
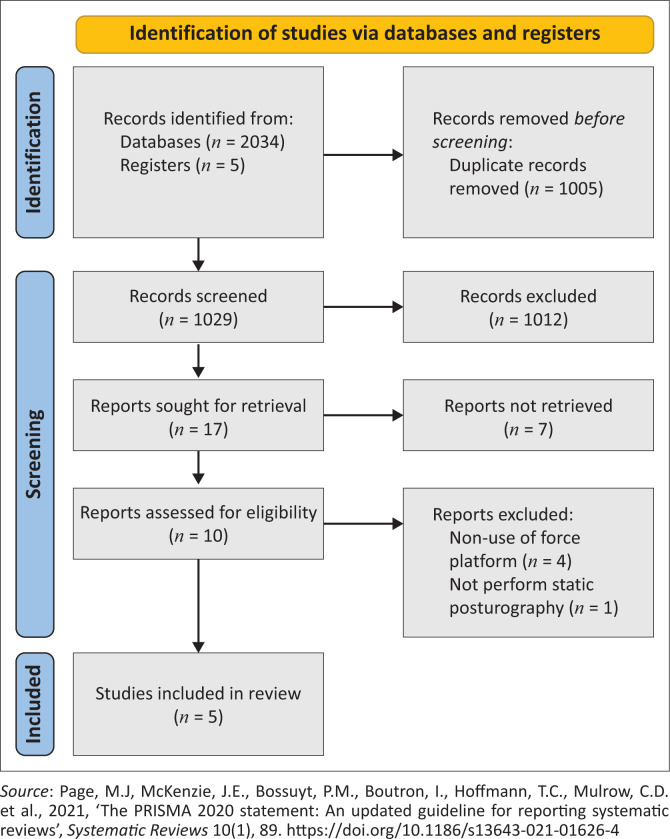
PRISMA flow diagram.

A total of 2034 studies were found by searching the databases (PUBMED = 688 studies; WEB OF SCIENCE = 254; EMBASE = 437; SCOPUS = 591; and SCIELO = 64). After excluding duplicate studies, 1029 remained, of which 1012 were excluded by title, leaving out 17 studies. Of this last value, 7 were excluded, leaving 10 studies for eligibility. Finally, of these 10 studies, 5 were excluded with justification (the research method did not classify them in the review) and 5 were included in our review, as follows: Bot et al. (1999), Sawacha et al. (2012), Vergara et al. (2012), De Nunzio et al. (2015) and Acar et al. (2019) (Figure 1).

### Participants

A total of 247 patients were included in the five studies, with sample sizes ranging from 15 to 70 individuals per study. Most of the studies were composed of men and women ranging in age from 18 to 77 years. Only two studies had only men as participants (Table 1).

**TABLE 1:** Description of included studies.

Author (year)	Sawacha et al. (2012)	De Nunzio et al. (2015)	Acar et al. (2019)	Vergara et al. (2012)	Bot et al. (1999)
**Aims**	To verify balance and posture and the relationship to AS.	To analyse postural control during static posture in patients with AS and to evaluate the effect of visual input to maintain posture.	To analyse the stability of the core musculature and the posture of individuals with AS compared to healthy individuals and to verify if there is any deficit in the aforementioned factors, in addition to investigating relationships between disease questionnaires and core stability and balance in AS patients.	To examine sagittal and frontal plane differences during static posturography in patients with AS compared to healthy subjects. In addition to evaluating the relationship between postural control measures and clinical disease questionnaires.	To analyse the possible mechanisms used to compensate for the anteriorization of the centre of mass in patients with AS.
**Sample size**	20 AS 20 controls	12 AS 12 controls	64 AS 64 controls	16 AS 17 controls	4 AS 18 controls
**Age of subjects**	$\bar{x}$ age AS = 51.4 years (SD = 13.9) and $\bar{x}$ control age = 49.4 years (SD = 10.4)	$\bar{x}$ age AS = 50.1 years (SD = 13.2) $\bar{x}$ age control = 43.5 (SD = 4.7)	$\bar{x}$ age AS = 39.9 (SD = 8.8) and $\bar{x}$ control age = 38.1 (SD = 9.7)	$\bar{x}$ age AS = 38.4 (SD = 12.5) and $\bar{x}$ control age = 36.4 (SD = 13.7)	AS age = 28–77 years and control age = 21–27 years
**Sex of the participants**	Men and women	Only men	Men and women	Men and women	Only men
**AS function and disease activity measures**	BASFI, BASDAI and BASMI	BASFI, BASDAI and BASMI	BASDAI, BASMI, ASDAS C and BASFI	BASDAI, BASMI, BASFI, ASQoL, patient global assessment and low back pain scores	None
**Platform**	Bertec	Baropodometric Platform (FDM-S)	Biodex Balance System	Two AMTI	AMTI
**Test duration**	60 s	50 s and 1 min break	Three attempts with a 10-s interval between each	Two attempts with 120 s each	Not described
**Posture and condition of patients**	Bare feet and 30 degrees apart, arms along the body, and eyes fixed on the target	Relaxed posture with arms besides the body. Bare feet 17 cm apart	Open eyes and bare feet	Bare feet, comfortable posture with feet apart, and arms besides the body. Informed to keep the gaze on a chosen spot on the wall	Not described
**Visual condition**	Eyes open and closed	Eyes open and closed	Eyes open	Eyes open and closed	Eyes open
**CoP variables analysed**	AP and ML displacement amplitude, area and total mean velocity	AP and ML displacement, total mean velocity, area and distance between consecutive points	Global, anteroposterior and mediolateral stability index	AP and ML displacement and area	AP and ML displacement
**Postural deviations in patients with AS**	Increased thoracic kyphosis and decreased anteversion, cervical and thoracolumbar ROM	Not described	Increased thoracic kyphosis and hip extension, deficit in trunk anterior and lateral flexion	Change in trunk anterior and lateral flexion	Increased thoracic kyphosis, knee flexion and plantarflexion
**Differences between control and AS in CoP variables**	Significant differences in ellipse with eyes closed, CoP extension in the ML direction with eyes open, total mean velocity in the eyes open condition, mainly in the ML direction (higher value for the AS group).	Differences in CoP displacement, total mean velocity, and AP and ML displacement amplitude (higher value for AS) and total displacement of sway (lower value for AS).	Changes in AS patients in stability in the AP and ML direction, unilateral (left) posture in the AP and ML direction and stability limits, except for the stability limit for the left.	Increase in total displacement of sway in the sagittal and frontal planes.	Hip ROM angle was lower in AS group and knee flexion angle and ankle plantarflexion were higher in this group.

ROM, range of motion; CoP, centre of pressure; AS, ankylosing spondylitis; ML, mediolateral; AP, anteroposterior; BASDAI, Bath Ankylosing Disease Activity Index; BASMI, Bath Ankylosing Spondylitis Metrology Index; ASDAS C, Ankylosing Spondylitis Disease Activity Score; BASFI, Bath Ankylosing Spondylitis Functional Index; ASQoL, Ankylosing Spondylitis Quality of Life Questionnaire; AMTI, Advanced Mechanical Technology, Inc.; FDM-S, Force Distribution Measurement - System.

### Equipment and collections

Among the included studies, most used force platforms (including a balance system), and only one used a baropodometric platform. Regarding the platform protocols, heterogeneity was found in the duration of the test and some similarities in the posture adopted and visual condition. Two of the studies had a test duration of less than 1 min, only one study had a duration of more than 1 min, and the remaining two did not report this information. Regarding the adopted posture, unanimously, the instructions given to the patients were standing bare feet, keeping the arms at the sides and staring at the target. Only two studies reported numerical values for the distance between the feet, and only one study (Sawacha et al. 2012) reported the distance between the platform and the target, which was 1 m. In the visual condition, three studies evaluated balance with eyes open and closed, and two of them performed only with eyes open.

### Centre of pressure variables

Most of the studies analysed CoP area, displacement and TMV. Only one study analysed the balance outcome with the stability index, which encompasses the global, AP and ML (Table 1). In nearly all studies, statistically significant differences were found between the postural control of patients with AS and apparently healthy individuals. Only one of the studies did not report the differences between AE (average entropy) and control for CoP variables. The main differences between the groups regarding the CoP were the greater displacement in the AP and ML directions, TMV, and area in patients with AS, indicating an increase in postural instability. Other differences in the intergroup CoP variables are highlighted in Table 1.

### Functional status

The physical function/quality-of-life assessment questionnaires used for patients with AS were BASFI (Bath Ankylosing Spondylitis Functional Index) (Calin et al. 1994), BASMI (Bath Ankylosing Spondylitis Metrology Index) (Jenkinson et al. 1994) and BASDAI (Bath Ankylosing Disease Activity Index) (Garrett et al. 1994). In addition to these questionnaires, one study included the ASDAS (Ankylosing Spondylitis Disease Activity Score) (Machado et al. 2011), and another added the ASQoL (Ankylosing Spondylitis Quality of Life Questionnaire) (Doward et al. 2003) and pain scores for the lower back. One of the studies did not report using any patient-reported outcome measure questionnaire. As regards postural deviations found after physical examination, three studies showed increased thoracic kyphosis, and two identified deficits during anterior and lateral trunk flexion. Other findings regarding postural deviations are presented in Table 1.

### Risk of bias analysis

For the analysis of the risk of bias, the cross-sectional study assessment tool AXIS (Downes et al. 2016) was used (Table 2). Only one study obtained a score of 100%, that is, it achieved all the requirements. Other studies obtained scores above 50% and only one was below that because of the lack of justification for the sample size, failure to choose a representative sample of patients with AS, failure to use disease-specific questionnaires, and failure to present the statistics adopted, initial data of the participants and study limitations. However, the main reason the studies did not obtain maximum scores was the lack of justification for the sample size.

**TABLE 2:** Risk of bias analysis of the cross-sectional study (AXIS).

Study		Bot et al. (1999)	Vergara et al. (2012)	Sawacha et al. (2012)	De Nunzio et al. (2015)	Acar et al. (2019)
**Introduction**						
(1)	Were the aims/objectives of the study clear?	Yes	Yes	Yes	Yes	Yes
**Method**						
(2)	Was the study design appropriate for the stated aim(s)?	Yes	Yes	Yes	Yes	Yes
(3)	Was the sample size justified?	No	No	No	No	Yes
(4)	Was the target/reference population clearly defined? (Is it clear who the research was about?)	Yes	Yes	Yes	Yes	Yes
(5)	Was the selection process likely to select subjects/participants that were representative of the target/reference population under investigation?	No	Yes	Yes	Yes	Yes
(6)	Were the risk factor and outcome variables measured appropriate to the aims of the study?	No	Yes	Yes	Yes	Yes
(7)	Were the risk factor and outcome variables measured correctly using instruments/measurements that had been trialled, piloted or published previously?	Yes	Yes	Yes	Yes	Yes
(8)	Is it clear what was used to determine statistical significance and/or precision estimates? (e.g. *P*, confidence intervals)	No	Yes	Yes	Yes	Yes
(9)	Were the methods (including statistical methods) sufficiently described to enable them to be repeated?	Yes	Yes	Yes	Yes	Yes
**Results**						
(10)	Were the basic data adequately described?	No	Yes	Yes	Yes	Yes
(11)	Were the results presented for all analyses described in the methods?	Yes	Yes	Yes	Yes	Yes
**Discussion**						
(12)	Were the authors’ discussions and conclusions justified by the results?	Yes	Yes	Yes	Yes	Yes
(13)	Were the limitations of the study discussed?	No	Yes	Yes	Yes	Yes
**Others**						
(14)	Were there any funding sources or conflicts of interest that may affect the authors’ interpretation of the results?	Uninformed	Yes	Yes	Yes	Yes
(15)	Was ethical approval or consent of participants attained?	No	Yes	Yes	Yes	Yes

## Discussion

Our systematic review of cross-sectional studies verified the characteristics of CoP variables during posturography in patients with AS compared to healthy individuals (control). Cross-sectional studies are particularly valuable for their ability to provide the characteristics of a population at a specific time point. This type of study is relevant in identifying the patterns and associations within a population, helping researchers gain insights into various aspects, from health outcomes to social behaviours. Moreover, observational studies often serve as a starting point for subsequent research, guiding the hypothesis and research question formulations for retrospective/prospective studies. The AXIS tool (Appraisal tool for Cross-Sectional Studies) was specifically created for evaluating cross-sectional studies and was designed to facilitate a systematic assessment and to help make informed critical judgements about its deductions.

Almost all studies included in our review presented a good score in the AXIS assessment. Vergara et al. (2012), Sawacha et al. (2012) and De Nunzio et al. (2015) received ‘yes’ to 14 of 15 items. However, Bot et al. (1999) poorly scored, with seven ‘no’ in its evaluation and one ‘undetermined’ item. The most frequent question negatively evaluated was about the justification of the sample size. According to Halpern, Karlawish and Berlin (2002) and Machin et al. (2008), the lack of sample size calculations and small sample sizes means that there is a low probability of finding a clinically relevant and statistically significant difference, which can lead to a high probability of inconclusive results. Only Acar et al. (2019) calculated the sample size, described the methods used, and received the maximum score on AXIS. Bot et al. (1999) reported some negative points such as no sample size justification, and the sample consisted of four patients; in the procedure, the exact time (in seconds) of the platform evaluation was not determined; one participant had total hip arthroplasty; descriptive statistical analysis; and absence of limitations in the Discussion section.

The presentation of CoP variables in the five included studies varied; hence, meta-analysis was not conducted. Vergara et al. (2012) found that displacement and frequency of the CoP in patients with AS are more affected in the frontal plane, ML direction, in relation to the sagittal plane, AP direction, and reported 49% increase in the root mean squared dispersion of the CoP shift when switching from eyes opened to closed, in agreement with Sawacha et al.’s (2012) results who found a greater extension of the CoP in the ML direction. Contrarily, De Nunzio et al. (2015) found a predominance of CoP displacement in the sagittal plane, AP plane and greater oscillation of the CoP in this same plane when the test was performed with eyes closed.

Another important CoP variable is the TMV. De Nunzio et al. (2015) reported a greater value for velocity with eyes closed in the AP direction when compared to eyes opened, which can be explained because the visual system greatly helps the individuals diagnosed with AS. Acar et al. (2019) showed a higher TMV, mainly in the ML direction, with eyes opened. No other studies have evaluated the TMV of the CoP. The stability limits of the AS group were analysed compared with the control group, which resulted in significant differences in all directions, except the left (Acar et al. 2019).

Displacement and frequency of oscillations in the sagittal and frontal planes, velocity in the AP and ML directions, area, total oscillation, displacement amplitude (AP and ML) and global stability index (AP and ML) were the most studied evaluated variables. Negative repercussions of the aforementioned variables on the AS group compared with the control group agree with studies published in the last two decades (Murray et al. 2000; Gunduz et al. 2017; Batur & Karatas 2017). Among these, the main differences noted were high scores for the displacement in the ML/AP direction and TMV and amplitude of the CoP in addition to the stability index, indicating a balance deficit. The anterior aspect could be explained due to stiffening and reduced mobility of the spine, pain, inflammation, muscle atrophy, enthesitis, possible osteoporosis and increased thoracic kyphosis, which were commonly found in the studies (Çinar et al. 2016; Murray et al. 2000; Masi et al. 2011). The clinical picture of the disease leads to a deficiency in the muscular and proprioceptive systems, which takes reduction in the sensory input together with muscle strength and control into consideration, the essential factors for maintaining the CG within the base of support (Batur & Karatas 2017; Yildirim & Yildirim 2015).

To maintain postural stability, patients generally assume a posture characterised by hip extension, knee flexion, ankle plantar flexion and posteriorisation of the pelvis. However, Sawacha et al. (2012) identified ankle dorsiflexion instead of plantarflexion, which was different from Bot et al.’s findings (1999). Other studies identified ankle dorsiflexion and plantarflexion to maintain postural control. Gokcen, Sariyildiz and Benlidayi (2021) reported altered foot postures, with reference to supination and pronation, in which a higher score was associated with supination, which was in turn related to plantarflexion (Seeger & Clarius 2009). In addition, these individuals exhibited fatigue and loss of muscle strength, especially in the quadriceps muscle (Masi et al. 2011), and changes in the anterior and lateral trunk flexion. This was reflected by changes in the muscle and ligaments of the abdominal region, such as muscle diastasis of the rectus abdominis, ossification of the ligaments and intramuscular fats (Grubisić et al. 2015), reduced activity of hip abductors and decreased muscle mass of the paraspinal muscle. However, Kim et al. (2017) reported no significant differences in the reduction of the muscle mass of the paraspinal muscle between patients and healthy individuals because this situation may be related to longer disease duration, which is characterised by chronic inflammation, increased cytokine levels and worsening postural changes (Resorlu et al. 2017).

Regarding the clinical characteristics and measures, patients with AS between 18 and 70 years were included in our study because some reports excluded individuals aged > 70 years as age is a relevant factor in postural balance. Only Bot et al. (1999) presented a sample of individuals aged > 70 years. Furthermore, the authors are composed of men and women, while some studies included only men because the disease is more prevalent in the male population (Wright et al. 2020).

Considering the visual condition in the tests, Bot et al. (1999) and Acar et al. (2019) did not perform the assessment under the eyes-closed condition, while Vergara et al. (2011) and De Nunzio et al. (2015) discussed the visual system and its relationship with respect to balance in individuals diagnosed with AS. These authors observed a greater dependence of patients on the visual system because an increase in the CoP displacement values was noted when passing from a static balance with eyes open to eyes closed, which is similar to the results of Günduz et al. (2017) and Çinar et al. (2016) Furthermore, Nogueira et al. (2020) investigated the posturography of patients with low back pain compared with healthy individuals and found muscle atrophy, intramuscular fat and proprioceptive changes in the patients with low back pain leading to greater dependence on vision, and therefore greater CoP displacement values with eyes closed. Therefore, this symptomatologic aspect strengthens the postural imbalance without vision. However, the visual system cannot sufficiently maintain the postural stability because, in the posture with eyes open, some values were lower than the normal range for the CoP variables (De Nunzio et al. 2015).

No standard duration of posturography tests has been established in the literature for these patients. Corriveau, Hebert & Prince (2004) analysed the reliability of CoP data using different durations of data collection and concluded that 120 s is the most reliable duration because of the non-stationary aspect of the CoP. However, Rugelj and Sevsek (2007) considered 30–60 s to be a safer duration because these patients have somatosensory deficits and are at greater risk of falls in attempts with longer durations.

Although researchers have investigated the postural control in individuals with musculoskeletal disorders, the results are still controversial. No standard CoP variables best represent balance, as the studies did not explore all the variables and provide little information about them. Therefore, further studies should be conducted with a better defined and comparable protocol so that different variables can be quantified, and dimensions of the loss of balance in these patients can be explored. In addition, further studies should discriminate which CoP variables can differentiate the group with the diagnosis and the control group.

### Limitations

The limitation of our study was the exclusion of the dynamic posture assessment on a force platform. Differences between the analysed CoP variables were also a limiting factor, which made it difficult to compare the studies. Still, only a few cross-sectional studies have evaluated the posturography of patients with AS compared with healthy individuals. Further studies are required to improve both the AS diagnosis and prognosis.

### Implications and recommendations

Individuals with AS presented postural instability and balance deficit. Therefore, exercises based on balance training and postural control should be considered in clinical practice. For scientific implications, new studies with unbiased protocols should be conducted, including appropriate sample calculations, standardisation of inclusion and exclusion criteria, and standardisation of the postural assessment protocol on the force platform. We suggest the evaluation time to be from 50 s, with a rest interval of 60 s between attempts. The following CoP parameters would be chosen: the TMV (cm/s) and the velocity in the AP (cm/s) and ML (cm/s) directions, which represent how fast the displacements were in both directions and in each direction, respectively; displacement amplitude in the AP (cm) and ML (cm) directions, representing the distance between the maximum and minimum displacements of the CoP in each direction; TOD (cm), which represents the total length of the CoP trajectory; and the area (cm^2^), which represents the displacement of the CoP within the ellipse (95%). In addition, data on ML dispersion (cm) and AP dispersion (cm) will also be analysed, referring to various amplitude data performed by the body in both directions.

## Conclusion

Our results reveal that patients with AS have a deficit in static posturography, especially in the eyes-closed condition. Therefore, CoP measurements are important variables to verify the evaluation of the patient in balance control during the treatment and disease progression.
